# Principles and limitations of NMR diffusion measurements

**DOI:** 10.4103/0971-6203.31148

**Published:** 2007

**Authors:** Jan Hrabe, Gurjinder Kaur, David N. Guilfoyle

**Affiliations:** Center for Advanced Brain Imaging, Nathan S. Kline Institute Orangeburg, New York, USA

**Keywords:** *b*-value, diffusion time, random walk, tortuosity, volume fraction

## Abstract

Diffusion spectroscopy, imaging and particularly diffusion tensor imaging have become popular thanks to their numerous clinical and research applications which span from brain stroke evaluation to fiber tracking. With a few exceptions, these methods are rooted in the classic Stejskal-Tanner formula for the diffusion-attenuated signal, usually obtained by solving the Bloch-Torrey partial differential equations. Here we derive the Stejskal-Tanner formula in the simplest possible manner, avoiding integrals and differential equations. This approach makes it easy to understand the origin of the diffusion signal attenuation, the effects of various diffusion sequence parameters, and also the numerous important pitfalls, which are discussed in the last section.

## Introduction

Most readers are probably aware of the diffusion tensor imaging (DTI) method,[1] which is ubiquitous in the contemporary medical MR literature. However, DTI is a relatively recent advance in diffusion experiments. NMR was used to measure diffusion long before MR imaging was first proposed in the early 1970s. The undesirable influence of self-diffusion on spin echo amplitudes was first recognized by Hahn as early as 1950.[2] Carr and Purcell[3] further extended Hahn's idea by using multiple echoes as a means of minimizing the diffusion effect. Shortly after these momentous discoveries, Torrey[4] extended the basic MR formalism of Bloch differential equations by including additional terms to accommodate the diffusion effect.

Stejskal and Tanner in their seminal paper[5] introduced pulsed gradients into the basic spin echo sequence, which resulted in much improved sensitivity to diffusion in comparison to the steady state gradients used previously. They solved the Bloch-Torrey partial differential equations for a symmetric pair of pulsed gradients and obtained the well-known Stejskal-Tanner formula

(1) $$S\;=\;S_{0}e^{-\mathrm{bD}},$$

where

(2) $$b\;=\;\gamma^{2}G^{2}\delta^{2}\left(\tau -\frac{1}{3}\delta \right)$$

In Eqs. (1) and (2), *S* is the signal strength in a pulse sequence with a pair of balanced diffusion-sensitizing gradients of strength *G*, each of a duration δ and with a delay τ between them. *S*_0_ is the signal strength in an identical experiment but without the diffusion gradient pair. When it can be safely assumed that δ ≪ τ, the expression for *b* (usually called *b*-value) simplifies to

(3) $$b={\text{γ}}^{2}G^{2}{\text{δ}}^{2}\text{τ}.\;$$

Tanner and Stejskal were also the first to propose the idea of measuring restricted diffusion of water molecules[6] by varying the delay τ between the gradient pulses. They discovered that the attenuation of signal from water molecules restricted by cellular walls in their diffusion movement was less pronounced than the signal from freely moving molecules and used this method to estimate the diameter of yeast cells.

Restricted diffusion plays a central role in DTI. Geometrically complex biological environments, such as the nervous tissue, can be characterized by two aggregate parameters,α and λ.[7] The volume fraction α is simply a percentage of the total tissue volume where molecules can diffuse. For example, in a healthy brain, the extracellular volume fraction is about 20% and the intracellular about 65%. The tortuosity parameter, λ, is more complicated.[8] It describes geometrical hindrance of an environment relative to an obstacle-free medium. If the obstacles exhibit some directional preference, the hindrance becomes anisotropic, that is, it depends on direction. For example, the molecules diffuse more readily along the white matter fibers than across them. In a macroscopically homogeneous and anisotropic environment, the tortuosity takes the shape of a symmetric tensor of the second order, which can be represented by a 3 × 3 matrix with six independent values. The tortuosity tensor combines with the scalar free diffusion coefficient into a tensor of apparent diffusion **D**. However, despite all this complexity, Eqs. (1) and (2) or (3) are still used to calculate signal attenuation due to diffusion along any single direction of the diffusion gradient. The only real difference from the homogeneous and isotropic case is that the experiment must be repeated using at least six non-collinear directions of the diffusion gradient to obtain six independent components of the apparent diffusion tensor. It is therefore fair to conclude that the pulse field gradient method proposed by Stejskal and Tanner is at the heart of most modern DTI experiments.

Unfortunately, there is an important caveat. A single imaging voxel in a nervous tissue often includes compartments with different diffusion properties both on a cellular level (e.g., intracellular and extracellular compartments) and on a larger scale (e.g., several crossing axonal bundles). Spins can also migrate from one compartment to another during the measurement. These complications violate the assumption of a macroscopically homogeneous and anisotropic environment with a single compartment. They may result in a non-Gaussian behavior of the diffusion signal for which the second order diffusion tensor no longer represents an adequate description.

DTI has become a very popular MR imaging modality and is developing into an important tool for non-invasive study and characterization of the brain white matter. It has been applied to the study of many neurological brain disorders such as schizophrenia, cocaine addiction, HIV infection, alcoholism, geriatric depression and Alzheimer's disease. An overview of theoretical issues surrounding the DTI technique can be found in.[9–11] A review of DTI applications in neuroscience is presented in.[12]

It is our view that good understanding of the Stejskal-Tanner formulae (Eqs. (1), (2) and (3)) is essential for sensible design or interpretation of any MR diffusion experiment. Unfortunately, the derivation in a completely general case is nontrivial as it involves either solving the Bloch-Torrey partial differential equations[4,5] or integration in a complex plane.[13,14] The goal of this report is to present a detailed derivation of Eqs. (1) and (3) using the simplest possible model of one-dimensional (1D) diffusion. While this approach lacks the complete generality afforded by the Bloch-Torrey description, it does preserve all important aspects of the physics involved and it does not require familiarity with higher mathematics beyond the binomial theorem and l'Hôpital's rule.

### The simplest diffusion model

**Figure 1:**
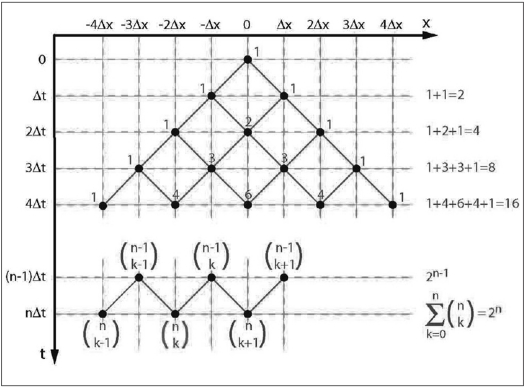
A 1D diffusion model with discrete steps in space (∆x) and time (∆*t*). The numbers signify how many pathways to that particular destination exist. Total number of all pathways is calculated on the right margin. See text for a detailed explanation.

This section reviews a connection between Gaussian diffusion and random walks under very simple circumstances. A comprehensive treatment can be found, e.g., in.[15] We shall consider a 1D diffusion of a particle, e.g., a hydrogen atom, along the *x*-axis. At time *t* = 0, the atom will be located at a position *x* = 0 [Figure 1]. We will allow it freedom of movement of a very limited kind: during every constant time interval ∆*t*, it may move either left or right, with equal probabilities *þ*_l_ = *þ*_r_ = $\frac{1}{2}$. The amount of movement is restricted to a jump of a constant size ∆*x*. It is obvious that the atom cannot get anywhere else than to positions *x* = 0, ±∆*x*, ±2∆*x*,…. We want to know the probabilities that it will be found at any of these positions at times *t* = 0, ∆*t*, 2∆*t*,….

Let us monitor the atom's progress step by step. Figure 1 shows that the atom is at position *x* = 0 at time *t* = 0 with certainty *P*(0, 0) = 1. Proceeding to time *t* = ∆*t*, there is one possible pathway to the left and one to the right, a total of two possibilities. The next time step is only slightly more complicated. At time *t* = 2∆*t*, the atom can be at one of three positions. A single pathway leads to each of the two peripheral positions but there are two different pathways (that is, two possibilities) leading to the center position. The number of pathways to the center position is equal to the sum of pathways for the previous positions from where the center position could be reached, that is, 1+1 = 2. The total number of all possible pathways is four.

The pattern of possible pathways is built up further in every time step. Counting the possible pathways, we end up with a number pattern called the Pascal triangle. Interestingly, these numbers also represent multiplication factors in the binomial theorem:

(4) $${\left(a+b\right)}^{n}=\sum_{k=0}^{n}\left(\frac{n}{k}\right)a^{n-k}b^{k}.$$

A general case at time *t* = *n*∆*t* is depicted in the lower part of Figure 1. There are ($\begin{array}{l} n\\ k \end{array}$) ways to get to a position placed *k* steps from the left-most position. The total number of all pathways grows by a factor of 2 at each time step. We can verify this observation using the binomial theorem, Eq. (4), with *a* = *b* = 1:

$\sum_{k=0}^{n}\left(\frac{n}{k}\right)1^{n-k}1^{k}\;=\;\sum_{k=0}^{n}\left(\frac{n}{k}\right)=\;{\left(1+1\right)}^{n}=2^{n}$

The probability *P*(*k*, *n*) of finding an atom at a position placed *k* steps from the extreme left edge after *n* time steps is calculated as a number of pathways leading to it, divided by the total number of all possible pathways:

(5) $$P\left(k,n\right)=\frac{1}{2^{n}}\left(\frac{n}{k}\right).$$

**Figure 2:**
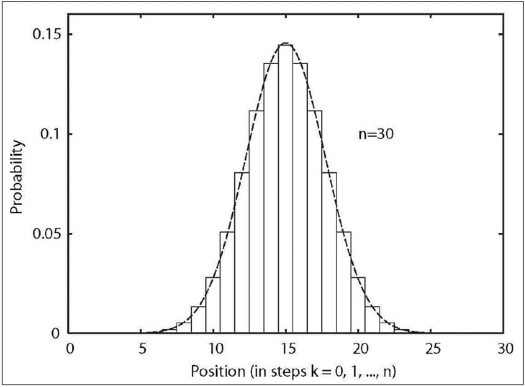
Discrete binomial distribution (*stepped line*) together with the corresponding continuous Gaussian distribution (*smooth line*). Note the similarity even for a very low *n* = 30. Indeed, these distributions are asymptotically identical.

This is a special case of the binomial distribution (also called Bernoulli distribution). The reason why the simple left-right jump pattern represents a fairly adequate 1D diffusion model rests in the central limit theorem of the probability theory, which mandates that the binomial distribution asymptotically approaches the Gaussian distribution if *n* becomes very large. The resemblance can already be seen for *n* = 30 in Figure 2.

Because the atoms move to the left and to the right with the same probability, they are equally likely to be on either side of the central position *x* = 0 at any time step. In other words, the mean value of the position is always

(6) <x>=0.

The mean position is therefore not very helpful in trying to estimate how far the atoms really moved because the positive and negative signs of the *x*-coordinate simply cancel out in the averaging. Instead, we can calculate the mean square distance from the origin, which is always positive. For our simple diffusion model, it can be obtained by mathematical induction: *n* = 1 at time *t* = ∆*t* and the distance from origin is either *x* = +∆*x* or *x* = -∆*x*. The average square of distance is then simply

${\text{σ}}^{2}\left(∆t\right)=<x^{2}\left(∆t\right)>=\frac{{\left(∆x\right)}^{2}+{\left(-∆x\right)}^{2}}{2}=∆x^{2}.$

Moving on to time *t* =(*n* - 1)*t*, we can calculate the effect of the *n*-th time step:

$\sigma^{2}\left(\mathrm{n∆t}\right)=\langle \frac{{\left(x\left(\left(n-1\right)\mathrm{∆t}\right)+\mathrm{∆x}\right)}^{2}\;+\;\left(x\left(\left(n-1\right)\mathrm{∆t}\right)\;-\;{\mathrm{∆x}}^{2}\right)}{2}\rangle \;\;=\langle x^{2}\left(\left(n-1\right)\mathrm{∆t}\right)+{\mathrm{∆x}}^{2}\rangle \;=s^{2}\left(\left(n-1\right)\mathrm{∆t}\right)+{\mathrm{∆x}}^{2}.$

It is clear that the mean square distance grows by the amount of ∆*x*^2^ in every time step:

${\text{σ}}^{2}\left(n∆t\right)=\langle x^{2}\left(n∆t\right)\rangle =n∆x^{2}.$

Index *n* represents progress of time and the mean square distance thus increases linearly with time. To make this relationship more explicit, we define a diffusion constant *D* as

(7) $$D=\frac{\text{∆}x^{2}}{2\text{∆}t}$$

and rewrite the equation for mean square distance in a more familiar form

(8) $${\text{σ}}^{2}\left(n\text{∆}t\right)=2Dn\text{∆}t.$$

This is the Einstein's famous formula for 1D diffusion in a “discrete form”. However, it is equally valid for a “continuous” 1D diffusion. A transition to continuous time and space variables can be made by taking smaller and smaller time steps ∆*t* and smaller and smaller spatial steps ∆*x* while taking care to preserve the value of the diffusion constant. This is easily arranged by always choosing

(9) $$\text{∆}x=\sqrt{2D\text{∆}t}.$$

The time interval ∆*t* then approaches zero and *n* goes to infinity in such a way that

t=n∆t

still shows the correct diffusion time. In this way, a generally valid result for Gaussian diffusion is finally obtained:

(10) $${\text{σ}}^{2}\left(t\right)=2Dt.$$

### The NMR diffusion signal

**Figure 3:**
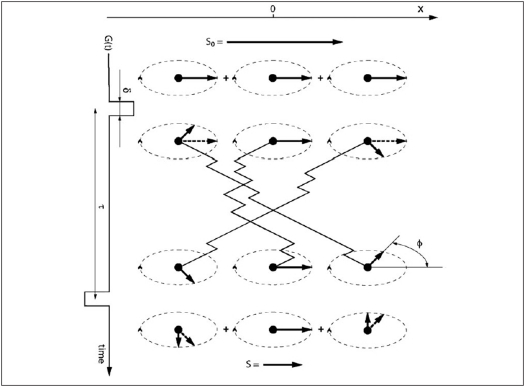
Nuclei diffusing in the presence of a balanced gradient pair *G* and −*G*. The two gradients are separated by a diffusion time interval *τ* and are very short (δ ≪ *τ*). Relaxation effects are omitted for simplicity. A diffusion gradient changes the phase of a spin depending on its position along the x-axis. *S*_0_ is the signal obtained without any diffusion gradients, *S* is the signal attenuated due to phase dispersion caused by the diffusion gradient pair and *ø* is the phase.

The principle of NMR diffusion measurement is depicted in Figure 3. Let us begin with a qualitative description of diffusion on the NMR signal. First, an RF pulse turns all the equilibrium magnetization *M*_0_ into the transverse plane, perpendicular to the main static magnetic field *B*_0_. The magnetization vector will rotate around it at angular frequency $\text{ω}=\frac{d\varphi }{dt}$ given by the Larmor equation:

(11) $$\text{ω}=\gamma B_{0},$$

where γ is the gyromagnetic ratio (γ =2π × 42.576 rad S^−1^ T^−1^ for a hydrogen proton).

After the excitation, a short and strong gradient *G* is applied along the *x*-axis, changing the constant main field *B*_0_ to a spatially variable field *B*(*x*) = *B*_0_ + *Gx*. Larmor frequencies therefore become different at different places along the *x*-axis. When the gradient is switched off again, some phase differences will have accumulated between the spins at different positions. At this point, we just wait for a period equal to the diffusion time τ = *n*∆*t*. Then an opposite but otherwise identical gradient, -*G*, is applied. If the atoms did not change their positions during the diffusion time, all the phase differences would be perfectly reversed and the magnetization would be fully restored (apart from the neglected relaxation effects). This is the situation exemplified by the center atom in Figure 3. However, if the spins move, the second gradient finds them at different locations than the first one and the phases will be reversed “incorrectly”. The result is a phase dispersion in the measured sample and loss of signal when all the spins are eventually summed up to form the magnetization vector. Faster diffusion (larger *D*) means that the spins have bigger chance to travel farther and therefore experience larger magnetic field changes due to diffusion gradients. This causes larger spread in the phases and therefore results in a smaller signal.

We can now proceed to a quantitative description and calculate the NMR signal using the simple diffusion model introduced earlier. The diffusion starts at time *t* = 0 from the position *x* = 0. At time *t* = *τ* = *n*∆*t*, the atom has undergone a total of *k* steps to the right and *k*_ steps to the left:

k−k_=n.

Its position measured in ∆*x* steps from *x* = 0 is therefore

$k_{x}=k-k\_=k-\left(n-k\right)=2k-n.$

The probability of finding it at *x* = *kx*∆*x* is given by Eq. (5), using the steps to the right (index *k*) to measure the position.

The atom located at *x* = *x*_0_ when the first gradient is switched on has its phase *ø* changed by

$∆\varphi_{0}=\text{ω}\left(x_{0}\right)\text{δ}=\text{γB}\left(x_{0}\right)\text{δ}=\text{γ}Gx_{0}\text{δ},$

where we took advantage of the Larmor Eq. (11) and assumed that the gradient time δ was very short so that the atom did not significantly move during the gradient application. The effect of the main field (γ*B*_0_δ) was left out because it is the same for any atom regardless of its position, its movement or the gradient strengths. During the diffusion time τ, the atom moves to a new position *x*_0_ + *k_x_*∆*x*. The second (negative) gradient then alters the phase again, this time by

${\mathrm{∆\varphi}}_{1}=\omega \left(x_{0}+k_{x}\mathrm{∆x}\right)\delta =\mathrm{\gamma B}\left(x_{0}+k_{x}\mathrm{∆x}\right)\delta =-{\mathrm{\gamma Gx}}_{0}\delta -{\mathrm{\gamma Gk}}_{x}\mathrm{∆x\delta}$

Assuming the phase was zero in the beginning (*ø* = 0 at *t* = 0), it is now, at *t* = τ,

$\varphi \left(k_{x}\right)={\mathrm{∆\varphi}}_{0}+{\mathrm{∆\varphi}}_{1}=-{\mathrm{\gamma Gk}}_{x}\mathrm{∆x\delta}$

Clearly, the result does not depend on the original position *x*_0_ and we can therefore safely return to the assumption *x*_0_ = 0.

To compute the NMR signal, it is necessary to sum up all the phase-shifted magnetization vectors from all possible positions using the corresponding probabilities as weighting factors. If the equilibrium magnetization is *M*_0_, the available signal *S* at *t* = *τ* is

(12) $$S=\sum_{k=0}^{n}\frac{1}{2^{n}}\left(\frac{n}{k}\right)M_{0}\;\cos\left(\varphi \left(2k-1\right)\right),$$

where we substituted *kx* = 2*k − n* for the phase function parameter.

This sum is best tackled using the Euler theorem

$\exp\left(\mathrm{i\alpha}\right)\;=\;\cos\left(\alpha \right)\;+\;\mathrm{isin}\left(\alpha \right)$

Abbreviating

α = -γG∆xδ

and employing again Eq. (4), we derive

$\begin{array}{ll} S & =\frac{M_{0}}{2^{n}}\sum_{k=0}^{n}\left(\frac{n}{k}\right)\exp\left(i\left(2k-n\right)\alpha \right)=\frac{M_{0}}{2^{n}\exp\left(in\alpha \right)}\sum_{k=0}^{n}\left(\frac{n}{k}\right)\exp\left(i2k\alpha \right)\\ & =\frac{M_{0}}{2^{n}{\left(\exp\left(i\alpha \right)\right)}^{n}}\sum_{k=0}^{n}\left(\frac{n}{k}\right){\left(\exp\left(i2\alpha \right)\right)}^{k}1^{n-k}\\ & =M_{0}{\left(\frac{\left(1+\exp(i2\alpha \right)}{2\exp\left(i\alpha \right)}\right)}^{n}=M_{0}{\left(\frac{\left(\exp(-i\alpha \right)+\exp\left(i\alpha \right)}{2}\right)}^{n}\\ & =M_{0}\cos^{n}\left(\alpha \right)=M_{0}\cos^{n}\left(\text{γ}G\text{δ}∆x\right). \end{array}$

The NMR signal undergoes various stages of amplification, filtering and other transformations. It is therefore not possible to measure *M*_0_ in absolute terms. We can remedy this unfortunate drawback of NMR by measuring the same sample twice: once without the diffusion gradients, to obtain unattenuated signal *S*_0_, and once with them, to obtain signal *S*. The *M*_0_ term in the signal will stay the same but the attenuation term will disappear from *S*_0_. We then calculate the ratio of the signals with and without the diffusion gradients:

$\frac{S}{S_{0}}=\frac{M_{0}\cos^{n}\left(\text{γ}G\delta ∆x\right)}{M_{0}}=\cos^{n}\left(\text{γ}G\delta ∆x\right).$

The last step is to eliminate the ∆*x* and express the result in terms of experimentally accessible variables. We substitute

$$\mathrm{\gamma G\delta ∆x}=\sqrt{\gamma^{2}G^{2}\delta^{2}{\mathrm{∆x}}^{2}}=\;\sqrt{2\left(\gamma^{2}G^{2}\delta^{2}\mathrm{n∆t}\right)}\frac{1}{n}\frac{{\mathrm{∆x}}^{2}}{2\mathrm{∆t}}=\sqrt{2\left(\gamma^{2}G^{2}\delta^{2}\tau \right)\frac{D}{n}}=\sqrt{\frac{2\mathrm{bD}}{n}},$$

where we introduced the so-called *b*-value,

(13) $$b={\text{γ}}^{2}G^{2}{\text{δ}}^{2}\text{τ},$$

a quantity which depends on the spectrometer hardware and the pulse program controlling it but does not depend on the diffusion constant. Note that the expression for the *b*-value becomes more complicated if various NMR sequence intricacies are taken into account. Most importantly, we assumed that the diffusion gradients are switched on for a negligible period of time in comparison with the diffusion time. However, Eq. (13) does capture the most important features—square dependency on the gradient moment *G*δ and linear dependency on the diffusion time *τ*.

To summarize, the NMR signal from nuclei following our simple diffusion model is attenuated due to phase randomization as

(14) $$\frac{S}{S_{0}}=\cos^{n}\left(\frac{\sqrt{2bD}}{n}\right).$$

This is a “discrete” form of the Stejskal-Tanner equation. Eq. (14) offers the possibility to measure the diffusion constant. This can be done by acquiring signals with and without diffusion gradients and calculating *D* from Eq. (14). Better still, one can obtain the signal many times with different *b*-values and obtain *D* by a fitting procedure.

### Continuous space and time

Almost everything is prepared for the continuous case in Eq. (14). It remains to make the discretization steps finer and finer to achieve, in the limit, a continuous change in space and time. For some constant diffusion time τ = *n*∆*t*, this is accomplished by simultaneously sending the ∆*t* to zero and *n* to infinity, while keeping the *D* constant. The space discretization step ∆*x* is always adjusted by Eq. (9) and is therefore also gradually diminishing towards zero.

In Eq. (14), this step refinement procedure simply means keeping *b* and *D* constant and calculating a limit for *n*→∞. The calculation will employ l'Hôpital's rule for limits of the $\frac{0}{0}$ type.

We first simplify

$\underset{n\to \infty }{\lim}\cos^{n}\left(\sqrt{\frac{2bD}{n}}\right)=\underset{n\to \infty }{\lim}e^{n\text{ ln }\left(\cos\left(\sqrt{\frac{2bD}{n}}\right)\right)}$

and concentrate on the limit in the exponent:

$\lim_{n\to \infty }\frac{\ln\left(\cos\left(\sqrt{\frac{2\mathrm{bD}}{n}}\right)\right)}{\frac{1}{n}}$

$\begin{array}{l} =\underset{n\to \infty }{\lim}\frac{{\left[\cos\left(\sqrt{\frac{2bD}{n}}\right)\right]}^{-1}\sin\left(\sqrt{\frac{2bD}{n}}\right)\frac{1}{2}\sqrt{2bD}n^{-\frac{3}{2}}}{-n^{-2}}\\ =-\sqrt{\frac{bD}{2}}\underset{n\to \infty }{\lim}\frac{\tan\left(\sqrt{\frac{2bD}{n}}\right)}{\frac{1}{\sqrt{n}}}\\ =-\sqrt{\frac{bD}{2}}\underset{n\to \infty }{\lim}\frac{{\left[\cos\left(\sqrt{\frac{2bD}{n}}\right)\right]}^{-2}\frac{1}{2}\sqrt{2bD}n^{-\frac{3}{2}}}{\frac{1}{2}n^{-\frac{3}{2}}}\\ =-bD\underset{n\to \infty }{\lim}\frac{1}{{\left[\cos\left(\sqrt{\frac{2bD}{n}}\right)\right]}^{2}}\\ =-bD. \end{array}$

The original limit can therefore be evaluated as

(15) $$\underset{n\to \infty }{\lim}\cos^{n}\left(\sqrt{\frac{2bD}{n}}\right)=e^{-bD}$$

and used to generalize Eq. (14) for continuous space and time by substituting Eq. (15) for its right side:

(16) $$\frac{S}{S_{0}}=e^{-bD}.$$

This is the Stejskal-Tanner formula, labeled as Eq. (1) in the introduction.

Practical application of Eq. (16) usually means taking its natural logarithm

(17) $$\text{ln}\left(\frac{S}{S_{0}}\right)=-bD.$$

Diffusion constant *D* is then extracted either directly from two experiments performed with and without the diffusion gradients or preferably by linear fitting of a series of signals acquired with different *b*-values.

Even though we derived the Stejskal-Tanner formula using a very simple 1D diffusion model, it is valid more generally and correctly describes NMR diffusion experiment in a homogeneous 3D environment as long as the diffusion behavior is Gaussian. Because this is not always the case, it is prudent to refer to the diffusion coefficient obtained from the Stejskal-Tanner formula as “apparent diffusion coefficient”.

In a 3D case, it is the diffusion gradient vector G = (*G_x_, G_y_, G_z_*) that determines the direction along which diffusion is measured. Diffusion perpendicular to the gradient vector does not alter the phase of magnetization in any way and is therefore invisible. Essentially then, NMR experiments measure a 1D diffusion along the diffusion gradient vector.

In an isotropic environment, application of a diffusion gradient along an arbitrary direction leads to the same signal attenuation predicted by the Stejskal-Tanner formula. In an anisotropic environment, at least six independent 1D diffusion measurements are required to fully assess the six independent components of the diffusion tensor. Each of these measurements still obeys the Stejskal-Tanner formula except that the diffusion coefficient *D* must be replaced by a scalar quantity

D=u·D̃·u,

where

$\text{u}=\frac{\text{G}}{\sqrt{G_{x}^{2}+G_{y}^{2}+G_{z}^{2}}}=\frac{\text{G}}{G}$

is a unit vector in the direction of the gradient, D is the diffusion tensor, and the dot signifies scalar multiplication. For example, in a coordinate system rotated to align u with the *x*-axis, *D* becomes the *D_xx_* component of the diffusion tensor D. Eq. (13) for the *b*-value remains valid regardless of the gradient direction or the coordinate system rotation, as long as the gradient pulses are much shorter than the diffusion time.

### Diffusion measurement pitfalls

In this last section, we endeavor to briefly point out some of the problems associated with NMR diffusion experiments. Particular emphasis is placed on brain diffusion measurements which have become very popular.

### Subject motion

Because diffusion sequences are so sensitive to even minute movement of spins, macroscopic sample motion is the biggest enemy of diffusion experiments.[16] Multi-shot sequences are virtually impossible to use in a living subject because even a very small motion affects the signal phase, making it difficult to combine the individual echoes into a consistent dataset. The usual answer comes in the form of single-shot echo planar imaging (EPI) which acquires the entire image in about 100 ms. Although the EPI method ensures much better phase consistency with respect to the diffusion gradients, it brings a slew of its own problems: low signal to noise ratio, low resolution and numerous distortions. Not even EPI sequences are completely immune to macroscopic motion, especially when the motion has variable velocity in the form of linear acceleration or rotation.[17]

### Imaging gradients

Although the diffusion gradient pair has dominant effect on the randomly walking spins, any other gradient present during the experiment also acts upon the magnetization phase and thus alters the diffusion signal. Besides the diffusion gradients, a typical MRI pulse sequence has a considerable number of other gradients used for slice selection, phase encoding, and readout. They can all act as additional and unwanted diffusion gradients. Their effect on the *b*-value can be estimated, given an accurate pulse sequence timing diagram.[18] This is important whenever a precise quantitative diffusion measurement is to be undertaken.

### Local gradients

Local magnetic field gradients due to inhomogeneities of magnetic susceptibility not only enhance the $T_{2}^{*}$ decay but also act as small spatially variable diffusion gradients. Unlike the pulse sequence gradients, the local gradients are usually not known in any detail and their precise effect therefore cannot be calculated. However, spin echo sequences decrease this type of diffusion weighting.[3] It can be shown that the *b*-value associated with the local parasitic gradients diminishes with a number of spin echoes squared.[19] Furthermore, alternation of the diffusion gradient polarity in a multi-echo sequence minimizes the effect of the background local gradients.[20]

### Dependence on the diffusion time

The result of a diffusion experiment very much depends on the diffusion time τ.[13] For example, if the diffusion time was extremely short, most molecules would not collide with any cellular walls or other obstacles and the process would closely approximate free diffusion. Longer diffusion time gives the molecules more opportunity to explore the complex cellular environment and sample its geometric tortuosity, as well as to move between the intracellular and extracellular compartments. Dependency on the diffusion time means that one cannot reasonably mix results obtained with different diffusion times. This also implies that the diffusion time should always be reported because data interpretation is not possible without it. Unfortunately, limited maximum gradient strength and the need to avoid eddy currents often result in relatively long diffusion gradients distributed throughout the sequence. It is impossible to precisely define the diffusion time of such sequences. Depending on the exact sequence design, such experiments can become essentially qualitative in nature and difficult to compare with those obtained by another sequence. The results acquired with such sequences would be truly quantitative only if the diffusion was strictly Gaussian, that is, if the diffusion constant (or tensor) did not depend on the diffusion time for the whole range of the diffusion times involved (e.g., in an agar gel phantom).

### Tissue compartments

The most common molecule used in NMR diffusion measurements is, by a large margin, water. Because water is ubiquitous in a living tissue, the detected signal typically comprises a mixture of components from the extracellular and intracellular compartments, generally of different diffusion properties.[21] There is a lively debate about their relative contributions under various circumstances, e.g., during ischemia.[22] The situation is considerably complicated by the exchange of water between these compartments.[23] The intracellular space may be compartmentalized even further due to the presence of organelles such as mitochondria.

### Inhomogeneous voxels

The voxel size in most diffusion imaging experiments, particularly in human imaging, is quite large in comparison to many brain features. However, the Stejskal-Tanner formula assumes constant *D* in the entire measured region. If the voxel contains both grey matter and white matter or if fiber tracts of different prevailing directions cross inside it, the assumption of homogeneity is violated. Similarly to the extracellular and intracellular contributions to the signal, the diffusion signal becomes a mixture of two or more compartments and the result will depend both on their individual properties and the rates of exchange between them.[24] However, the voxel partial volume effects can also be useful, aiding the diffusion-based tissue segmentation.[25] The degree to which diffusion is non-Gaussian can be quantified with diffusional kurtosis imaging.[26] For example, if the voxel environment comprises a number of separate compartments, each with its own Gaussian diffusion, kurtosis imaging can establish the degree of diffusion coefficient variability inside this voxel.

### Apportioning the DTI imaging time

Many brain regions, most notably the white matter tracts, are significantly anisotropic. As we discussed earlier, the Stejskal-Tanner formulae can still be used in this case but the experiment has to be repeated with different gradient directions. There is a considerable body of literature on the optimum number and arrangement of the gradient vectors used.[9–11] However, one should keep in mind that the optimum choice depends to a large degree on the subject under study and thus varies from one type of experiment to another. For example, larger anisotropy may require more sampling directions. In general, given a finite available imaging time, the tradeoff is made by spending it on more signal averages, more gradient directions, more different *b*-values, or more different diffusion times. There is no simple and universal answer to this puzzle.

### Temperature and viscosity

Diffusion depends not only on the size of the diffusing molecule (its Stokes hydrodynamic radius) and on geometric tortuosity of the environment but also on temperature and viscosity. Viscosity in turn also depends on temperature. The overall effect is a faster diffusion at higher temperatures.[27,28] This dependency has to be taken into account, e.g., when comparing human *in-vivo* data with *in-vitro* samples examined at room temperature.

### Larger molecules

It is perfectly feasible to measure diffusion of other substances than water. Standard MR spectroscopic techniques can be used, with addition of diffusion gradients.[28] The attenuation of signal peaks corresponding to the individual metabolites still obeys the Stejskal-Tanner formulae. However, smaller gyromagnetic ratios of these substances are reflected in smaller *b*-values (see Eq. (13)). The only remedy is to use larger diffusion gradients. Furthermore, because efficient signal detection requires that the exponent *bD* does not become too small, larger and slower metabolites require even larger *b*-values to compensate for their lower diffusion coefficient and are therefore particularly affected.

### Scanner hardware

Diffusion sequences are very demanding on the scanner. The gradient set is required to perform very accurately and achieve perfect symmetry of the diffusion gradient pairs.[16] Equally important is their spatial linearity. Because the *b*-value depends on a square of the gradient, a nonlinearity of 5% over the subject head will translate into 10% error in diffusion coefficient. Suppression of eddy currents is also essential. They frequently produce observable artefacts quite capable of ruining the diffusion experiment. Careful pulse sequence design[29] and various post-processing corrections[30,31] certainly help in this respect but the most important factor is the quality of the scanner hardware and its maintenance. Good performance on standard clinical sequences in no way guarantees that the same system can be successfully used for diffusion experiments.

The list presented above may create a somewhat pessimistic impression regarding the ability of the NMR diffusion method to obtain truly quantitative results, particularly in living nervous tissue. However, despite all the problems, much valuable and relevant information has been extracted by their employment. Clearly, good understanding of the Stejskal-Tanner formulae is crucial to avoid suboptimal experimental designs and misinterpretations of results.
